# Understanding the role of intimate partner violence on HIV transmission in Zimbabwe: Secondary data analysis of data from the Zimbabwe demographic survey 2015-2016

**DOI:** 10.34172/hpp.2023.14

**Published:** 2023-07-10

**Authors:** Munyaradzi Mapingure, Tafadzwa Dzinamarira, Zindoga Mukandavire, Innocent Chingombe, Diego F. Cuadros, Rouzeh Eghtessadi, Farirai Mutenherwa, Helena Herrera, Roda Madziva, Solomon Mukwenha, Grant Murewanhema, Godfrey Musuka

**Affiliations:** ^1^ICAP at Columbia University, Harare, Zimbabwe; ^2^School of Health Systems and Public Health, University of Pretoria, Pretoria, South Africa; ^3^Emirates Aviation University, Centre for Data Science and Artificial Intelligence, Dubai, UAE; ^4^Department of Geography and Geographic Information Science, University of Cincinnati, Cincinnati, USA; ^5^SAfAIDS Regional Office, Harare, Zimbabwe; ^6^Biomedical Research & Training Institute, Harare, Zimbabwe; ^7^Portsmouth University, Portsmouth, United Kingdom; ^8^School of Sociology and Social Policy, University of Nottingham, Nottingham, United Kingdom; ^9^Unit of Obstetrics and Gynecology, Faculty of Medicine and Health Sciences, University of Zimbabwe, Harare, Zimbabwe

**Keywords:** HIV, Risk factors, Zimbabwe, Gender-based violence

## Abstract

**Background:**

Gender-based violence (GBV) has been shown to have significant and long-lasting impacts on women’s physical and mental health. It is, therefore, important to study its occurrence in a population and its intersect with infectious diseases such as HIV to inform the wider health promotion agenda. This study aimed to determine the association between GBV and HIV status in women and adolescent girls in Zimbabwe.

**Methods:**

A secondary data analysis of data from a cross-sectional Zimbabwe Demographic and Health Survey (ZDHS) was conducted. Statistical analysis was employed to establish the association between GBV and HIV status. Geospatial mapping was conducted using a kernel smoothing method was employed to generate a continuous kernel density surface to illustrate the local spatial variations of female HIV and GBV prevalence.

**Results:**

Women and adolescent girls suffering emotional GBV, such as those subjected to humiliation by their husbands or partners, were 1.45 (1.14-1.84) [OR (95% CIs)] times more likely to be HIV positive than those who were never humiliated. The same was true for women and adolescent girls whose husbands or partners threatened to harm them or someone they love, 1.33 (1.04-1.68). There is a relationship between women’s HIV status and intimate partner aggression, such as when their partners pushed, shook, or threw something at them or physically abused them. This was also the case for those who reported that partners kicked, dragged, or beat them, tried to choke or burn them on purpose, or threatened or attacked them with a knife, gun, or other weapons. Women who experienced forced sexual violence with threats were more likely 1.61 (1.08-2.41), to be HIV positive than those women who did not experience the same.

**Conclusion:**

GBV is widely spread in Zimbabwe. There is a need for the government to implement creative strategies to reach out to survivors, especially those that are forced to have unprotected sex and are at increased risk of HIV acquisition. This manuscript raises issues that can be addressed by robust health promotion strategies to reduce the impact of the syndemic of GBV and HIV acquisition in Zimbabwe.

## Introduction

 Zimbabwe bears one of the worst HIV burden globally with an HIV prevalence of 14% among the general public,^1^ with 1 300 000 million individuals living with HIV.^2,3^ Despite a constant decline over the past decade,^4,5^ adolescents, mainly girls, remain a priority group disproportionately affected by HIV in Zimbabwe. This pattern is observed in other African countries. Therefore, research on the role of gender inequalities in marriage and sexual relationships and their contribution to HIV infection^6,7^ has been of critical importance with the recognition that the elimination of any form of violence against women and adolescent girls is an important component of the HIV response and vital for sustainable development.^8^

 Gender-based violence (GBV) against women and adolescent girls is a gross violation of human rights and is also widely recognised as fuelling HIV transmission.^9,10^ For instance, an HIV model that assessed the effects of gender-based inequalities in Southern Africa revealed that gender-inequality-induced reproductive number was greater than the reproductive number in the absence of gender-inequality.^9^ Cultural and traditional defined gender roles and norms that define what is acceptable behaviours also play a critical role. In Zimbabwe’ societies, gender roles and identities often define males as aggressive and females as submissive, contributing to ineffective sexual negotiation skills for females. This deepens power imbalances between females and males.^11,12^ Such norms are likely to increase sexual GBV, resulting in increased vulnerability to HIV, other sexually transmitted infections, and unplanned pregnancies.^13^

 Victim blaming and stigmatization and the threat of partner violence often deter women and adolescent girls from revealing their HIV status to their partners or seeking HIV services. Studies have showed thata considerably high proportion of GBV occurs within the marriage institution and other intimate relationships.^14^ A growing body of literature exists on the prevalence and trends of domestic violence in Zimbabwe.^15^ For example, it is documented that violence against women and adolescent girls cuts across the socio-economic, religious, and cultural divide.^15^ Furthermore, trends in the prevalence of GBV against women and adolescent girls have shown very little change over the past 15 years.^15^ 2006, 36% of women and adolescent girls aged 15-49 who experienced physical violence since age 15 were HIV positive. In 2011, the HIV prevalence in the same group fell from 36% in 2005-206 to 30%. However, a sharp increase to 35% was reported in 2015.^15^ During pregnancy, violence against women and adolescent girls decreased slightly from 8% in 2005-2006 to 5% in 2010-2011 and 6% in 2015.^15^ In all three-national demographic and health surveys conducted between 2005 and 2015, the current husband or partner was invariably reported as the main perpetrator of the violence. Estimates from the Zimbabwe Demographic and Health Survey (ZDHS) showed that 14% and 8% of adolescent girls aged 15-49 reported having ever experienced sexual violence, and having experienced this form of violence within the past 12 months of the survey respectively.^15^ As with physical violence, the current husband was reported as the perpetrator. The experience of physical or sexual violence was highest among women aged 25-29 (48%).

 GBV undermines the dignity, autonomy and self-esteem of the victims; and the overall social and economic development of the entire society, hereby often re-enforcing gender in-equalities. GBV has been shown to have significant and long-lasting impacts on physical and mental health including injury, and pregnancy complications on women. It is important to study its occurrence in a population and its intersect with infectious diseases such as HIV to inform the wider health promotion agenda. This study investigates the association between GBV and female HIV status by performing a secondary analysis of the 2015-2016 ZDHS data. Additionally, it uses geospatial mapping to show the distribution of GBV and areas of high HIV prevalence. The geospatial mapping will assist the government of Zimbabwe in identifying areas with the highest concentration of the GBV and HIV syndemic so that appropriate targeted programmes can be implemented in the locations with the greatest need.

## Materials and Methods

###  Study area and data sources

 The data was obtained from the ZDHS. The ZDHS was a cross-sectional survey conducted nationwide. The survey employed a two-stage sampling procedure for household selection. For this secondary data analysis, the study population was 5291 women and adolescent girls aged 15 to 49 years who were ever in a marital union, and responded to the violence module of the survey and received an HIV test. Trained data collectors administered the survey questionnaire in English Shona or Ndebele language. Vironostika Uniform 2 Ag/AB enzyme-linked immunosorbent assay (ELISA) and the Enzygnost® HIV Integral II assay (Siemens) were employed for HIV testing with a positive classification resulting from both tests being reactive. DiaSorin, an HIV western blot assay was the tie breaker in cases where the first two tests were discrepant.

 The data was obtained from the ZDHS, a nationwide cross-sectional survey.

###  Statistical analysis

 STATA version 15.1 (StataCorp. 2017. Stata Statistical Software: Release 15. College Station, TX: StataCorp LLC, Texas USA) was used to analyse the data statistically. This began by using descriptive statistics to describe the characteristics of the participants. Associations between GBV variables and HIV status were explored using the chi-square test for categorical variables. We also calculate odds ratios (ORs) and their 95% confidence intervals (CIs) for HIV positivity among women and adolescent girls experiencing various forms of GVB compared to those who did not. The statistical significance cut-off for describing the significant GBV factors associated with HIV positivity was set at *P* < 0.05. To represent the geographical structure of HIV and the prevalence of each GBV variable in the sample and to identify potential areas where these variables had similar spatial distribution, maps of the HIV prevalence and GBV variables reported were produced using the geographic coordinates for each primary sample unit (PSU) where the survey was conducted. First, the prevalence of HIV in females was estimated at each PSU. HIV prevalence (*HIVp*) at PSU location *i* was defined as HIVp_i_ = *n*_i_*/ N*_i_, where *n*_i_ denotes the number of HIV-positive females and *N*_i_ denotes the total number of females at location *i.* The prevalence of each category of GBVat each PSU was calculated similarly to the HIV prevalence. The number of female participants with “yes” responses was divided by the total number of responders at each PSU. A kernel smoothing method was employed to generate a continuous kernel density surface to illustrate the local spatial variations of female HIV and GBV prevalence.^16^

## Results

 Data from5291 women and adolescent girls aged 15 years and above was included in the study. The mean age was 31.5 and standard deviation was 8.0. These had a definitive HIV status and had also completed the GBV module of the ZDHS. Most of them (64% of HIV positive and 66% of HIV negative) were residents in rural areas. Regarding educational attainment, the majority (60% of HIV positive and 63% of HIV negative) had completed secondary education, while 1% of the HIV positive and 2% of HIV negative had none. Regarding marital status, the majority (64% among the HIV positive and 85% among the HIV negative) were married. When grouped by religious affiliation, the two dominant groups were apostolic (44% among the HIV positive and 46% among the HIV negative) and pentecostal (24% among the HIV positive and 23% among the HIV negative) sects, as shown in Table 1.

**Table 1:** Baseline characteristics of female respondents to the 2015-2016 ZDHS

**Variable **	**HIV Positive, ** **No. (%) (n=1135)**	**HIV Negative, ** **No. (%) (n=4156)**
Age group (y)		
15-19	16 (1)	267 (7)
20-24	110 (10)	807 (18)
25-29	183 (16)	908 (22)
30-34	270 (24)	889 (22)
35-39	235 (21)	573 (14)
40-44	218 (20)	437 (11)
45-49	103 (9)	275 (6)
Type of residence		
Urban	475 (36)	1657 (34)
Rural	660 (64)	2499 (66)
Highest education level		
None	10 (1)	59 (2)
Primary	382 (35)	1126 (29)
Secondary	684 (60)	2658 (63)
Higher	59 (4)	313 (7)
Marital status		
Married	716 (64)	3527 (85)
Living with partner	73 (6)	167 (4)
Widowed	157 (13)	108 (3)
Divorced	116 (11)	190 (5)
Separated	73 (6)	164 (4)
Religion		
Traditional	2 (0)	31 (1)
Roman catholic	74 (7)	250 (6)
Protestant	155 (14)	593 (14)
Pentecostal	282 (24)	1023 (23)
Apostolic sect	463 (44)	1796 (46)
Other Christian	60 (4)	235 (4)
Muslim	7 (1)	9 (0)
None	90 (7)	215 (6)
Other	2 (0)	4 (0)

Abbreviations: HIV, human immunodeficiency virus; ZHDS, Zimbabwe Demographic and Health Survey.

 GBV was categorised into three broad types in this paper: emotional, physical, and sexual. Below are findings under each of the three GBV categories. Overall, the prevalence of emotional, physical, and sexual violence was 32%, 32%, and 13%.

 As shown in Table 2, the odds of being HIV positive were higher for women and adolescent girls who experienced various forms of emotional GBV than those who did not. For example, women and adolescent girls who were ever humiliated by their husbands or partners were 1.45 times more HIV positive than those never humiliated, *P* = 0.002. The same was true for women and adolescent girls whose husbands or partners threatened to harm themselves or someone they love, OR (95% CI) 1.33 (1.04-1.68), *P* = 0.022. However, the results were insignificant for women and adolescent girls whose husbands or partners ever insulted or made them feel bad about themselves.

**Table 2:** Association between GBV variables and HIV status

**Variable **	**HIV Positive, No. (%)**	**HIV Negative, No. (%)**	**Unadjusted OR (95% CI)**	***P*** ** value**
**Emotional violence (Did your (last) husband/partner ever do the following?)**			
Said or did something to humiliate in front of others			
No	958 (19.2)	3679 (80.8)		
Yes	177 (25.6)	477 (74.4)	1.45 (1.14-1.84)	0.002
Threatened to hurt or harm or someone you care about			
No	980 (19.4)	3686 (80.6)		
Yes	155 (24.2)	470 (75.8)	1.33 (1.04-1.68)	0.022
Insulted or made to feel bad about self.			
No	831 (20.0)	3067 (80.0)		
Yes	304 (20.1)	1089 (79.9)	1.01 (0.84-1.22)	0.921
**Physical violence (Did your (last) husband/partner ever do the following)**			
Push you, shake you, or throw something at you?			
No	976 (19.1)	3739 (81.0)		
Yes	159 (28.3)	417 (71.7)	1.67 (1.31-2.14)	0.001
Slap you?			
No	821 (20.1)	3075 (79.9)		
Yes	314 (19.9)	1081 (80.1)	0.99 (0.82-1.19)	0.906
Twist your arm or pull your hair?			
No	1061 (19.8)	3942 (80.2)		
Yes	74 (24.2)	214 (75.8)	1.30 (0.92-1.83)	0.142
Punch you with his fist or with something that could hurt you			
No	987 (19.3)	3767 (80.7)		
Yes	148 (25.9)	389 (74.1)	1.46 (1.14-1.86)	0.002
Kick you, drag you, or beat you up?			
No	1014 (19.6)	3811 (80.4)		
Yes	121 (25.0)	345 (75.0)	1.37 (1.05-1.78)	0.021
Try to choke you or burn you on purpose?			
No	1090 (19.7)	4064 (80.3)		
Yes	45 (31.4)	92 (68.6)	1.86 (1.16-2.99)	0.01
Threaten or attack you with a knife, gun, or any other weapon?			
No	1094 (19.7)	4076 (80.3)		
Yes	41 (34.2)	80 (65.8)	2.12 (1.35-3.31)	0.001
**Sexual violence (Did your (last) husband/partner ever do the following)**			
Physically force you to have sexual intercourse with him even when you did not want to?			
No	1018 (19.7)	3781 (80.3)		
Yes	117 (22.7)	375 (77.4)	1.19 (0.91-1.56)	0.209
Physically force you to perform any other sexual acts you did not want to?			
No	1039 (19.9)	3833 (80.1)		
Yes	96 (21.5)	323 (78.5)	1.10 (0.82-1.48)	0.516
Force you with threats or in any other way to perform any sexual acts you did not want to?			
No	1084 (19.7)	4034 (80.3)		
Yes	51 (28.4)	122 (71.6)	1.61 (1.08-2.41)	0.019

Abbreviations: HIV, human immunodeficiency virus; CI, confidence interval; OR, odds ratio; GBV, Gender-based violence. *Note*: We used chi-square tests to compared the various dichotomous variables between HIV positive and HIV negative participants.

 Physical violence was also significantly associated with having an HIV-positive status. As shown in Table 2, results show an association between HIV positivity and women and adolescent girls who reported various forms of GBV. These included their husband or partner pushed, shook, or threw something at them or punched them with a fist or with something that could hurt them or kicked, dragged, or beat them, or tried to choke or burn them on purpose, or threatened or attacked them with a knife or gun, or any other weapon.

 Women and adolescent girls who experienced sexual GBV were more likely (OR 1.61, CI: 1.08-2.41, *P* = 0.019) to be HIV positive than those women and adolescent girls who did not experience this (Table 2). However, there was no significant association between forced sexual intercourse or other sexual acts and HIV infection. Results for multivariate analysis are shown in Table 3 where having a partner who drinks alcohol as associated with higher HIV positivity adjusted OR, 1.38 (1.18-1.62), *P* < 0.001.

**Table 3:** Multivariate analysis of Association between GBV variables and HIV status

**Variable**	**HIV positive, No. (%)**	**HIV negative, No. (%)**	**Unadjusted OR (95% CI)**	***P *** **value**	**Adjusted OR (95% CI)**	***P *** **value**
Age (y)						
Mean (SD)	34.3 (7.3)	30.7 (8.1)	1.06 (1.05-1.07)	0.001	1.06 (1.05-1.07)	0.001
Highest education level						
None	10 (13)	59 (87)	1		1	
Primary	382 (23)	1126 (77)	2.01 (0.99-4.11)	0.054	2.81 (1.36-5.78)	0.005
Secondary	684 (19)	2658 (81)	1.59 (0.78-3.23)	0.198	2.24 (1.09-4.60)	0.029
Higher	59 (14)	313 (86)	1.09 (0.50-2.38)	0.826	1.37 (0.61-3.08)	0.44
Area of residence						
Urban	475 (21)	1657 (79)	1		1	
Rural	660 (20)	2499 (80)	0.91 (0.78-1.08)	0.281	0.71 (0.51-0.99)	0.042
Wealth Index						
Poorest	212 (20)	742 (80)	1		1	
Poorer	171 (19)	687 (81)	0.91 (0.72-1.16)	0.47	0.90 (0.70-1.15)	0.401
Middle	192 (21)	667 (79)	1.02 (0.80-1.29)	0.893	0.98 (0.76-1.26)	0.863
Richer	340 (23)	1123 (77)	1.17 (0.94-1.47)	0.169	1.00 (0.71-1.40)	0.994
Richest	220 (17)	937 (83)	0.81 (0.63-1.03)	0.09	0.63 (0.41-0.97)	0.036
Partner drinks alcohol						
No	586 (18)	2525 (82)	1		1	
Yes	549 (24)	1631 (76)	1.41 (1.21-1.64)	0.001	1.38 (1.18-1.62)	0.001
Emotional violence						
No	772 (20)	2861 (80)	1		1	
Yes	363 (20)	1295 (80)	0.98 (0.83-1.15)	0.785	0.88 (0.73-1.07)	0.199
Physical violence						
No	768 (20)	2899 (80)	1		1	
Yes	367 (21)	1257 (79)	1.09 (0.92-1.28)	0.312	1.13 (0.94-1.36)	0.201
Sexual violence						
No	987 (20)	3668 (80)	1		1	
Yes	148 (21)	488 (79)	1.08 (0.86-1.36)	0.485	1.10 (0.86-1.40)	0.463

Abbreviations: HIV, human immunodeficiency virus; CI, confidence interval; OR, odds ratio; SD, standard deviation; GBV, Gender-based violence.

**Figure 1:**
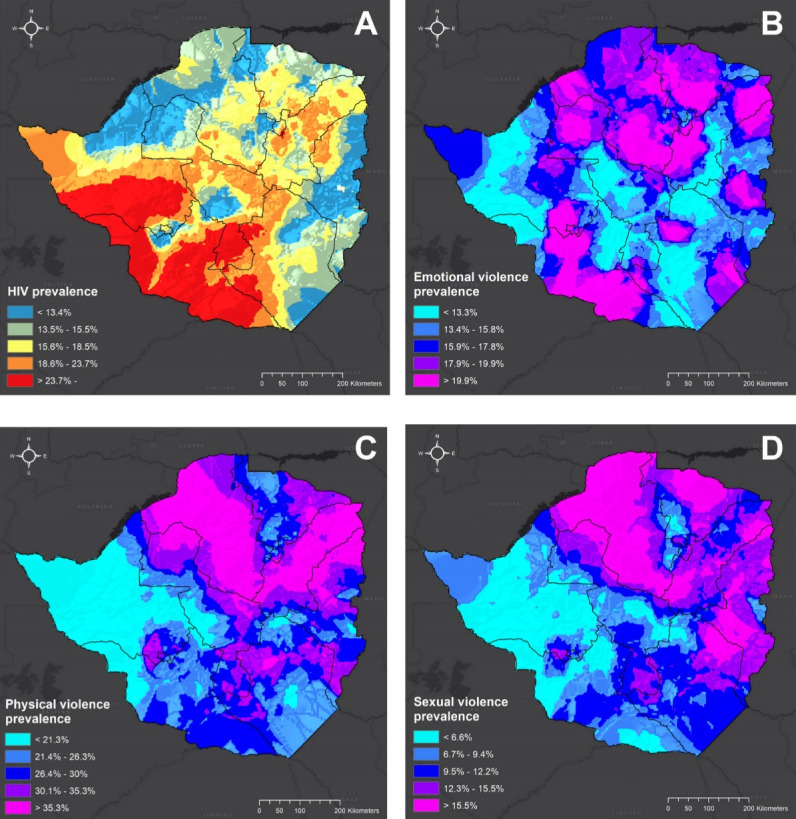
The spatial distribution of (A) female HIV prevalence; (B) emotional violence prevalence; (C) physical violence prevalence; and (D) sexual violence prevalence. Maps were created using ArcGIS^®^ by ESRI version 10.5 (http://www.esri.com ).^17^

 Geospatial analysis showed that all forms of GBV (emotional, physical and sexual) are endemic across Zimbabwe and occur in every part of the country to a substantial degree. In terms of distribution, the high prevalence of the three categories of GBV is distinctly concentrated in the provinces of Mashonaland West, Central and East, and Harare, in the northern part of the country. Additionally, only emotional GBV had similar spatial distribution as the high HIV prevalence distribution in females, particularly in the southern part of the country, within Matabeleland North and South (Figure 1).

## Discussion

 Our study results showed that the different categories of GBV (emotional, physical, and sexual) are prevalent across Zimbabwe. There is a significant association between various forms of violence and HIV status. Studies have demonstrated that as women’s empowerment decreases, the likelihood of inter-partner violence increases. For example, an analysis of the 2010-2011 ZDHS data^10^ showed that women and adolescent girls aged 15-49 years who did not participate in decision-making at a household level were more likely to experience GBV than those who did. Further, those who had some control over their spouses’ earnings were less likely to suffer from GBV. Economic factors may contribute to GBV, which subsequently exposure them to HIV infection or reinfection.

 For example, women and adolescent girls may be forced to engage in transactional sex for survival and are unable to negotiate safe sex due to economic dependence on a casual or long-term partner who provides financial security for herself, her family, or siblings; as well as child marriage.^10^ Using a feministic framework to understand the association between inter-partner violence and HIV status in Zimbabwe, Amowitz LL et al found that married and cohabitating women and adolescent girls who experienced various forms of violence from their partners were more likely to be HIV positive compared to those who did not.^11^ In addition, the study also demonstrated that adolescent girls with controlling partners were also more susceptible to HIV.

 GBV against women and adolescent girls can lead to adverse health outcomes.^12-14^ Additionally, 23% of females believed that women should not ask their partners to use condoms if they had a sexually transmitted infection.^15^ It would be critical to explore how women who experience GBV cope with their HIV status, uptake of HIV medication, and viral load in this context of a male-centred society to guide evidence-based prevention and care programmes.

 Our geospatial analysis confirms the high prevalence of forms of GBV and their distribution across the country.^18^ Programme managers could use microplanning methods to identify geographic locations that require priority attention and what aspects further action should focus on. For any initiative to succeed, there is a need for robust and sustained engagement of key community stakeholders, such as religious, traditional, and cultural leaders, alongside Government officials to sensitise them to the reach and negative impact of GBV. Champions to empower women and adolescent girls to achieve further perceived rights and better health should be recruited at the local level to create a conducive environment for reporting and addressing all forms of GBV.

 The findings of this study aim to contribute to a better understanding of the HIV epidemic in Zimbabwe and GBV as one of its contributing factors and catalyse further research on this topic. It would be particularly timely when interlinkage exists between two epidemics (GBV and HIV), both prone to worse due to the economic and humanitarian impact of a further epidemic caused by COVID-19.

## Limitations

 This work’s cross-sectional study design does not enable the establishment of causality between variables and outcomes. In addition, social desirability may have affected the provision of responses to the ZDHS, resulting from these questionnaires administered by an interviewer.

## Conclusion

 GBV is prevalent in Zimbabwe and is associated with increased risk of forced unprotected sex which may lead to HIV acquisition. Zimbabwe will benefit from HIV prevention interventions that integrate screening for GBV into strategic service delivery points, such as HIV testing services (HTS) or pre-exposure prophylaxis (PrEP) services, to support the provision of treatment within public sector facilities, and to facilitate specific referral mechanisms to other support services for GBV survivors.

## Acknowledgements

 The authors would like to thank all respondents for their willingness to participate in the ZDHS study and for ICF Macro for making the data available.

## Competing Interests

 All authors declare that they have no competing interests.

## Ethical Approval

 Procedures and questionnaires for standard Demographic Health Surveys (DHS) have been reviewed and approved by the ICF International Institutional Review Board (IRB). Additionally, the ICF IRB reviewed country-specific DHS survey protocols, typically by an IRB in the host country. The ICF International IRB ensures that the survey complies with the U.S. Department of Health and Human Services regulations for protecting human subjects. In contrast, the host country IRB ensures that the study complies with the laws and norms of the nation. In the original primary data collection for each DHS, informed consent was sought from all participants before serological testing for HIV (http://dh.sprogram.com/What-We-Do/Protecting-the-Privacy-of-DHS-Survey-Respondents.cfm#sthash.Ot3N7n5m.dpuf ). We sought and were granted permission to use the core dataset for this analysis by MEASURE DHS. For minors, informed consent was obtained from their parents or guardians.

## Funding

 This study was not funded.

## References

[1] Zimbabwe National Statistics Agency (ZIMSTAT), ICF International. Zimbabwe Demographic and Health Survey 2015: Final Report. Rockville, MD: ZIMSTAT, ICF International; 2016.

[2] UNAIDS. Country Factsheets: Zimbabwe 2016. Available from: http://www.unaids.org/en/regionscountries/countries/zimbabwe.

[3] Ministry of Health and Child Care (MoHCC). Zimbabwe Population-Based HIV Impact Assessment (ZIMPHIA 2020): Final Report. Harare: MoHCC; 2021.

[4] Gonese E, Mapako T, Dzangare J, Rusakaniko S, Kilmarx PH, Postma MJ (2015). Within-gender changes in HIV prevalence among adults between 2005/6 and 2010/11 in Zimbabwe. PLoS One.

[5] Sibanda E, Khumalo P (2017). A review of interprovincial variations in HIV prevalence rates in Zimbabwe. Afr J AIDS Res.

[6] Jewkes R (2010). Jewkes RHIV/AIDSGender inequities must be addressed in HIV prevention. Science.

[7] Duffy L (2005). Culture and context of HIV prevention in rural Zimbabwe: the influence of gender inequality. J Transcult Nurs.

[8] Kusuma YS, Babu BV (2017). Elimination of violence against women and girls as a global action agenda. J Inj Violence Res.

[9] Mukandavire Z, Malunguza NJ, Chiyaka C, Musuka G, Tchuenche JM (2010). HIV/AIDS model assessing the effects of gender-inequality affecting women in African heterosexual settings. Int J Biomath.

[10] Dunkle KL, Jewkes RK, Brown HC, Gray GE, McIntryre JA, Harlow SD (2004). Transactional sex among women in Soweto, South Africa: prevalence, risk factors and association with HIV infection. Soc Sci Med.

[11] Amowitz LL, Reis C, Lyons KH, Vann B, Mansaray B, Akinsulure-Smith AM (2002). Prevalence of war-related sexual violence and other human rights abuses among internally displaced persons in Sierra Leone. JAMA.

[12] Pradhan MR, Ram U (2010). Perceived gender role that shape youth sexual behaviour: evidence from rural Orissa, India. J Adolesc.

[13] Lekalakala-Mokgele E (2016). Exploring gender perceptions of risk of HIV infection and related behaviour among elderly men and women of Ga-Rankuwa, Gauteng province, South Africa. SAHARA J.

[14] Devries KM, Mak JY, García-Moreno C, Petzold M, Child JC, Falder G (2013). Global health. The global prevalence of intimate partner violence against women. Science.

[15] GBD 2017 HIV Collaborators (2019). Global, regional, and national incidence, prevalence, and mortality of HIV, 1980-2017, and forecasts to 2030, for 195 countries and territories: a systematic analysis for the Global Burden of Diseases, Injuries, and Risk Factors Study 2017. Lancet HIV.

[16] Carrat F, Valleron AJ (1992). Epidemiologic mapping using the “kriging” method: application to an influenza-like illness epidemic in France. Am J Epidemiol.

[17] ESRI. ArcGIS 10.x. Redlands, CA: ESRI; 2004.

[18] Emmanuel S, Jephias M, John B, Tendai S, Manford G (2022). In their own voices-understanding GBV in Zimbabwe: evidence from a survivors perspective. J Afr Stud Dev.

